# Formation of Stereocomplex Crystal and Its Effect on the Morphology and Property of PDLA/PLLA Blends

**DOI:** 10.3390/polym12112515

**Published:** 2020-10-28

**Authors:** Xiaolong Su, Lihua Feng, Demei Yu

**Affiliations:** School of Chemistry, State Key Laboratory of Electrical Insulation and Power Equipment, Xi’an Jiaotong University, Xi’an 710049, China; suxl1117@stu.xjtu.edu.cn (X.S.); fenglihua@mail.xjtu.edu.cn (L.F.)

**Keywords:** polylactic acid, stereocomplex, dielectricity, mechanical properties

## Abstract

Stereocomplex-polylactic acid (SC-PLA) is obtained in poly(d-lactic) acid/poly(l-lactic) acid (PDLA/PLLA) blends under adjusting processing conditions. It is found that the degree of crystallinity of overall SC-PLA is up to 43.7% in PDLA/PLLA blends of 1:1 mass ratio. Formation of stereocomplex (SC) crystals forces molecular chains in the blends to be more closely arranged and further enhances interaction between molecular chains, thus forming a physical cross-linking network in the SC crystals, resulting in the blends having a special microstructure. The mechanism of formation of the SC crystal physical cross-linking network is elucidated by dielectric spectroscopy, and the relationships between homocomplex (HC) crystals, SC crystals, and amorphous regions in the blends are also analyzed. Interestingly, mechanical properties of the blends are significantly improved due to formation of an SC crystal cross-linking network.

## 1. Introduction

In recent years, increasing consumption of petroleum-based materials and production of plastic waste has resulted in severe environmental pollution, and addressing this issue is critical. Polylactic acid (PLA) is a common biodegradable thermoplastic polyester that is widely used in delivery systems, 3D printing, biobased packaging, and other fields, owing to its excellent biocompatibility, processability, and environmental friendliness [1,2,3,4]. In addition, PLA has three different structures, namely, poly(l-lactic) acid (PLLA), poly(d-lactic) acid (PDLA), and poly (dl-lactic) acid (PDLLA). Among these, PDLA and PLLA can be combined with each other to form stereocomplex-polylactic acid (SC-PLA), which was first reported by Ikada et al. [5]. Compared with PLLA and/or PDLA, SC-PLA has a higher melting point of 210–230 °C, and its mechanical properties, e.g., thermal stability, are also significantly improved. This is because the two isomers (PDLA and PLLA) are in the form of complementary helical structures in SC-PLA, and the unique hydrogen bond (C=O^…^H_3_C) and dipole–dipole interaction between the two isomers optimizes the properties of SC-PLA [6]. This special structure leads to formation of stereocomplex (SC) crystals in SC-PLA.

Notably, during SC crystal formation in blending of PDLA and PLLA, some homocomplex (HC) crystals are also formed by PLLA and/or PDLA. Compared with HC crystals, formation of SC crystals is more difficult because their diffusion paths are lengthy, and the diffusion process is restricted [7]. Additionally, SC crystals that are formed first can also induce formation of HC crystals. Therefore, to avoid HC crystal formation in PLA, many research groups have directed their efforts toward studying processing technology for preparing PLA. Yamamoto et al. prepared SC-PLA with a high molecular weight and crystallinity by repeated solution casting [8]. In the process of repeated dissolution and casting, the SC crystals initially formed do not dissolve, and only HC crystals undergo dissolution. With an increase in repetition cycles, the content of SC crystals gradually increases until almost no HC crystals remain in the blends. The SC-PLA without HC crystals can also be prepared by controlling melting temperature or annealing temperature during blending of PDLA/PLLA. Zhang et al. annealed amorphous PDLA/PLLA blends and obtained blended films with a high content of pure SC crystals [9].

Study of internal structures and crystallization behaviors of the materials by broadband dielectric spectroscopy, a popular characterization method, has been extensively reported in recent years. Fujita et al. performed a comprehensive analysis of pure PLLA using dielectric spectroscopy by varying spherulite size and annealing conditions of pure PLLA. The crystallization behavior of pure PLLA at different temperatures and the interaction between the amorphous region and crystal region in pure PLLA were analyzed using the technique [10,11]. In addition, some researchers introduce polymers, nanoparticles, or ionic liquids into PLA systems to improve the properties of the blends and analyze the mechanism using dielectric spectroscopy [12,13,14]. However, there are only a few reports describing crystallization behavior and the internal structure of SC-PLA using broadband dielectric spectroscopy.

In this study, PDLA/PLLA blends with different PDLA contents were prepared via a melt-blending method using a Haake Torque rheometer. In order to obtain the overall SC-PLA, a special processing temperature range (190–220 °C) was selected for melt-blending during the processing. The microstructure, crystallization behavior, and dielectric and mechanical properties of the PDLA/PLLA blends were investigated and the mechanism of the formation of SC crystals was educed.

## 2. Materials and Methods

### 2.1. Materials

The PLLA (grade 4032D) was purchased from Nature Works LLC (Blair, NE, USA). Its number average molecular weight (Mn) was 1.6 × 10^5^ g/mol and polydispersity (PDI) was 1.7. PDLA was purchased from Jinan Daigang Biomaterial Co., Ltd. (Jinan, China). The Mn and PDI of PDLA were 1.53 and 1.7 × 10^5^ g/mol, respectively. Trichloromethane (analytical grade) was purchased from Tianjin Fuyu Fine Chemical Co., Ltd. (Tianjin, China).

### 2.2. Preparation of the Blends

PDLA and PLLA particles were dried overnight in a vacuum at 40 °C, and PLLA and PDLA/PLLA blends were prepared using a Haake Torque rheometer (Rheocord 9000, Thermo Fisher Scientific, Waltham, MA, USA). The processing temperature range and speed of screw were 190–220 °C and 15 r/min, respectively. The PDLA contents in the total masses of PDLA/PLLA blends were 1%, 2%, 5%, 10%, 20%, 30%, 40%, and 50%, respectively. Extruded PDLA/PLLA blends were cooled to room temperature (20 °C) and then a sample was cut into particles using an electrically controlled granulator (JD1A-90, LvDao Ltd., Suzhou, China). The particle samples were hot-pressed at 230 °C for 15 min using a plate vulcanizing machine (QLB-25T, QunLong Ltd., Xiamen, China) and then cooled to room temperature (20 °C). The obtained film samples and specimens were used for dielectric and mechanical properties tests.

### 2.3. Characterization and Performance Testing

#### 2.3.1. Crystallization Behavior

The melting/crystallization process of pure PLLA and PDLA/PLLA blends was characterized using a differential scanning calorimeter (DSC, Q100, Berne, Switzerland), employing a heating/cooling rate of 10 °C/min in the temperature range of 30–250 °C in purified nitrogen atmosphere. DSC measurements were performed from 30 to 250 °C at a heating rate of 10 °C/min, melted at 250 °C, and kept for 3 min. They were then cooled to 30 °C at a cooling rate of 10 °C/min, kept at 30 °C for 3 min, and reheated to 250 °C at a heating rate of 10 °C/min for the second heating run. In order to eliminate the influence of the thermal history of the sample on the experimental results, DSC test data are the results of the second melting process. Ten milligrams of samples were used to ensure instrument sensitivity. The degree of crystallinity, which is a key parameter of the blends, is closely related to other properties of the blends. The degree of crystallinity of PLLA and/or SC-PLA (*X*_c_) was determined using Equation (1):

(1) $$X_{c}\%=\frac{\Delta H_{m1}+\Delta H_{m2}-\Delta H_{c}}{\Delta H_{m(blend)}^{0}}\times 100$$

where ∆*H*_m1_ and ∆*H*_m2_ are the melting enthalpies of PLLA and SC-PLA, respectively, and ∆*H*_m0(*blend*)_ (106 or 142 J/g) [15,16,17] is the melting enthalpy of 100% crystalline of the PLLA or SC-PLA.

X-ray diffraction (XRD) patterns of pure PLLA and PDLA/PLLA blends were obtained using a D8 diffractometer (Bruker D8 Advance, Bruker Corporation, Dresdon, Germany) in the diffraction angle range of 2θ = 5°–30°, with a scan speed of 5°/min at 20 °C.

#### 2.3.2. Morphology and Microstructure Observation

The crystalline morphology of the pure PLLA and PDLA/PLLA blend crystals were observed using a polarizing microscope (POM, Bx51, Olympus Corporation, Tokyo, Japan) with a magnification of 200× or 500×, and the samples were placed on a hot plate with a heating rate/cooling rate of 10 °C/min in the temperature range of 30–250 °C.

The microstructure of the extrusion was investigated. The extruded wire samples of both PLLA and PDLA/PLLA blends were immerged in liquid nitrogen for 30 min and then were fractured by breaking. Fracture surfaces were observed using field-emission scanning electron microscopy (FE-SEM) (Gemini SEM500, Carl Zeiss AG, Oberkochen, Germany). The phase domain distribution of the PDLA/PLLA blends and the morphology of SC crystals were observed using transmission electron microscopy (TEM) (JEM-2100, JEOL Ltd., Tokyo, Japan). The SC crystal sample was obtained by dissolving the overall SC-PLA in 0.1% trichloromethane solution.

#### 2.3.3. Testing of Dielectric and Mechanical Properties

The dielectric properties of PDLA/PLLA blends and pure PLLA were tested using a Novocontrol broadband dielectric spectrum test system (Novocontrol Technologies, Montabaur, Germany). Before the test, the 1 × 25 mm^2^ (thickness × diameter) film samples were dried in a vacuum at 40 °C for 24 h, and then the front and back of the samples were sputtered using a particle sputtering apparatus (MC1000, HITACHI Ltd., Tokyo, Japan) containing a round gold electrode with a diameter of 22 mm. The applied bias voltage was 1 V, the test temperature range was −20–120 °C, and the test frequency range was 10^−1^–10^6^ Hz.

Tensile tests were performed employing an electromechanical universal testing machine (WDW-5H, TuoBo Ltd., Suzhou, China). At 20 °C, the samples were stretched at a crosshead speed of 20 mm/min with a load cell of 5 kN in accordance with ISO-527-2. Each sample was tested at least five times.

## 3. Results and Discussion

### 3.1. Crystallization of the Blends

**Figure 1:**
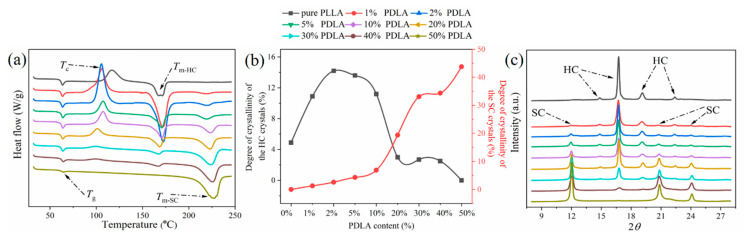
(**a**) DSC curves, (**b**) degree of crystallinity, and (**c**) XRD data of pure poly(l-lactic) acid (PLLA) and poly(d-lactic) acid (PDLA)/PLLA blends with different PDLA contents.

To understand the formation of SC crystals in the blends, the melting/crystallization process and crystal types of the pure PLLA and PLLA/PDLA blends with different PDLA contents were examined by DSC and XRD, respectively. As shown in Figure 1a, pure PLLA exhibits only one melting peak (melting peak of HC crystals) at 171.2 °C. After introduction of 1% PDLA, a new low intensity peak is observed at 219.1 °C, corresponding to the melting peak of SC crystals. With an increase in PDLA content, the melting peak of SC becomes more noticeable, and the melting temperature of SC crystals also increases gradually. When the PDLA and PLLA mass ratio is 1:1, only the melting peak of SC crystals is observed at 227.7 °C, while the melting peak of HC crystals disappears completely. Therefore, it can be shown that the overall SC-PLA can be successfully prepared in the PDLA/PLLA blends of 1:1 mass ratio by the above melt-blending method. In addition, the cold-crystallization peak of PLLA is about 116.8 °C, while the cold-crystallization peak of the blends is about 105.7 °C. It is 100.9 °C when the PDLA content is 1% and 20%, respectively. The shift toward low temperature indicates that introduction of PDLA can improve the crystallization capacity of PDLA/PLLA blends. Cold-crystallization peaks of the blends disappear gradually when the PDLA content increases continuously to 30–50%, indicating transition of PDLA and PLLA domains to the SC-PLA domain.

Notably, the area of melting peak of HC crystals first increases and then decreases, and the corresponding area of cold-crystallization peak also first increases and then decreases until it completely disappears. This is consistent with the variation trend of the degree of crystallinity of HC crystals determined using Equation (1), as shown in Figure 1b. It is considered that a small amount of SC crystals could act as a nucleating agent to promote crystallization of HC crystals. However, when PDLA content is >10% of the total mass of the blends, the physical cross-linking network in SC crystals can inhibit crystallization of HC crystals, resulting in a decrease in the degree of crystallinity of HC crystals. Additionally, the degree of crystallinity of SC crystals increases with an increase of PDLA content, and the degree of crystallinity of the overall SC-PLA is up to 43.7% when PDLA content reaches 50%.

XRD data of pure PLLA and PDLA/PDLA blends with different PDLA contents are shown in Figure 1c. The curve of pure PLLA shows four diffraction peaks at 14.8°, 16.7°, 19.1°, and 22.4°, which are the characteristic diffraction peaks reflected by the HC crystals [18]. When the doping amount of PDLA is 1%, three new diffraction peaks are observed at 11.9°, 20.7°, and 24.0°, corresponding to diffraction peaks reflected by the SC crystals [19]. It is indicated that even a small amount of PDLA can form SC crystals with PLLA. With an increase in PDLA content, the diffraction peak of SC crystals becomes sharp and increases in intensity, while the diffraction peak of HC crystals decreases in intensity until its complete disappearance. The results of XRD analysis further confirm that the overall SC-PLA is successfully prepared using the above melt-blending method.

### 3.2. Morphology and Microstructure of the Blends

**Figure 2:**
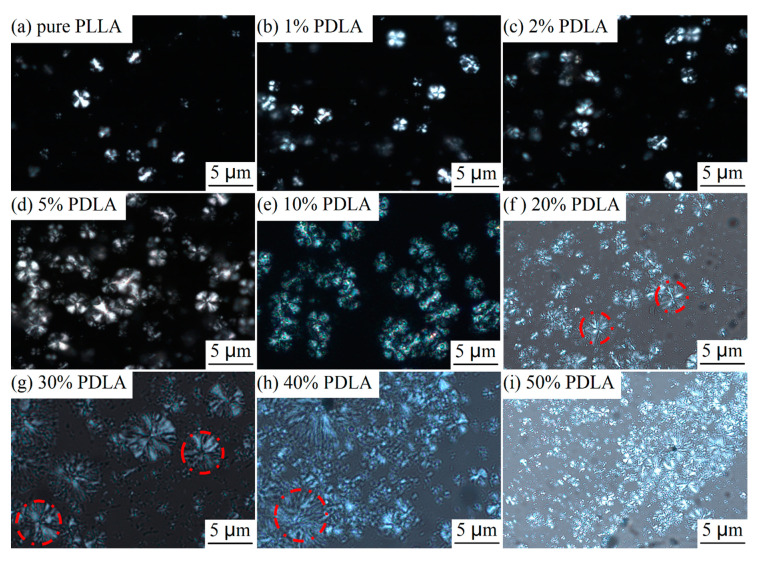
POM images of (**a**) pure PLLA and (**b**–**i**) PDLA/PLLA blends with different PDLA contents.

The morphology of crystals can provide insights into the formation process of crystals in the blends. As shown in Figure 2a, the morphology of pure PLA crystals is spherulite with the Maltese cross, and the spherulite diameter is approximately 10 μm. After introduction of PDLA, spherulite density increases significantly, which shows that addition of PDLA causes an increase in the number of nuclei (nucleation seeds), thus improving the crystallization capacity of the blends. When PDLA content is >10%, most spherulites decrease in size, while a small amount of spherulites increases in size, as shown in the red circles in Figure 2f–h, which is caused by the co-existence of HC crystals and SC crystals.

**Figure 3:**
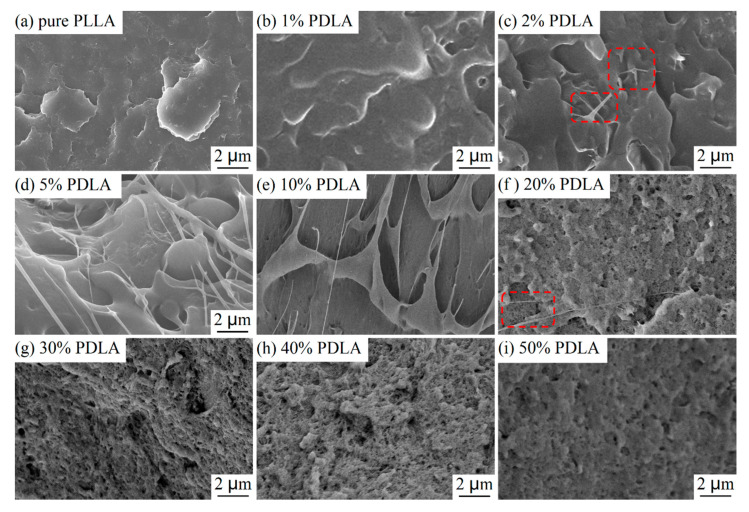
FE-SEM images of the morphologies of extrusions of (**a**) pure PLLA and (**b**–**i**) PDLA/PLLA blends with different PDLA contents.

To understand the multiphase microstructure of the blends, the cross-section of extrusions of PDLA/PLLA blends fractured under liquid nitrogen is observed and the experiment results are shown in Figure 3. It is found that cross-section characteristics of PDLA/PLLA blends can be divided into three kinds. When PDLA content is <5%, cross-sections of PDLA/PLLA blends do not change significantly compared with that of pure PLLA, which is because most of the components in blends with low PDLA content are still amorphous. Among them, a small number of filamentous burrs are formed on the cross-section of extrusions of blends with PDLA content of 2%, as shown in the red rectangles in Figure 3c, indicating that the microstructure of blends has begun to change. When PDLA content is between 5% and 10%, filamentous burrs are observed on the cross-section of the PDLA/PLLA blends. Subsequently, the amount of SC crystals increases in blends with increased PDLA content, resulting in disappearance of burrs on cross-sections of the blends. Only a few filamentous burrs remained on the cross-section of extrusions of blends with PDLA content of 20%, as shown in the red rectangles in Figure 3f. A special microstructure of the blends appeared, as shown in Figure 3f–i. To explore this special microstructure, the phase domain distribution of the components of the PDLA/PLLA blend with a mass ratio of 1:1 was observed by TEM.

**Figure 4:**
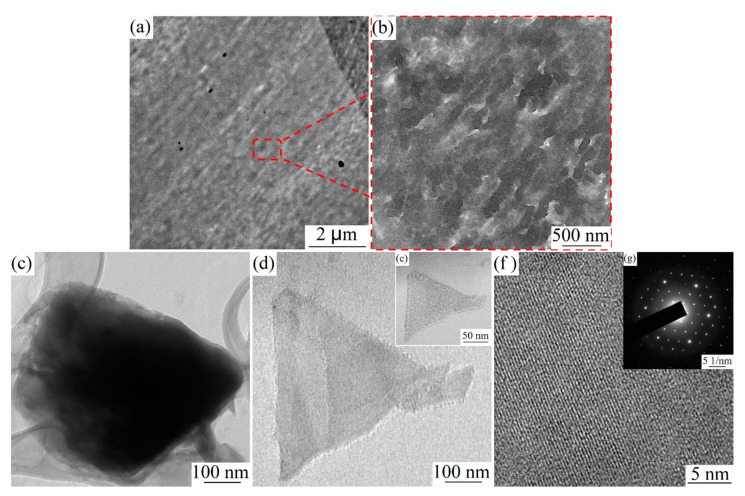
TEM images of (**a**,**b**) 50% PDLA/PLLA blends, (**c**–**e**) SC lamellar crystals, and (**f**,**g**) corresponding electron diffraction patterns of stereocomplex (SC) crystals.

It was found that the interface region division of PDLA and PLLA is not noticeable, as shown in Figure 4a,b, indicating formation of overall stereocomplex-polylactic acid (SC-PLA). Furthermore, the morphology of SC crystals in SC-PLA is detected. The SC crystals were obtained by dissolving the PDLA/PLLA blend with a mass ratio of 1:1 in trichloromethane. Their morphology is shown in Figure 4c–e. It is found that the SC lamellar crystal is a triangular microstructure. From the orientation relation between the electron diffraction and crystal morphology, respective sides of this triangular crystal correspond to (110), (120), and (210) planes, as shown in Figure 4f–g.

### 3.3. Mechanism for the Formation of the Cross-Linking Network in the SC Crystals

**Figure 5:**
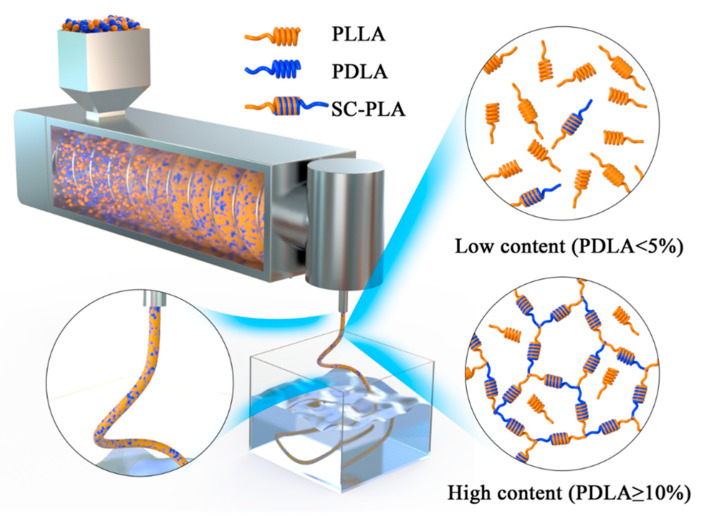
Mechanism for formation of a cross-linking network of SC crystals.

From investigation of the morphology and microstructure of the PDLA/PLLA blends, as mentioned above, the mechanism of the special structure of SC-PLA is inferred. As shown in Figure 5, the SC-PLA formed by a small amount of PDLA in PLLA is insufficient to form a cross-linking network in the blends. In addition, SC crystals are well separated in the blends and act as a nucleating agent to increase the degree of crystallinity of PLLA. In contrast, for blends with high PDLA content (PDLA ≥ 10%), the amount of SC-PLA crystals formed increases gradually and reaches a physical cross-linked network, combining the unique hydrogen bond and dipole–dipole interaction between the two isomers. The cross-linking network formed in the SC crystals can transform the molecular chain from a linear structure to a branched cross-linked structure, resulting the special microstructure of the blends, as shown in Figure 3.

### 3.4. Dielectric Behaviors of the Blends

**Figure 6:**
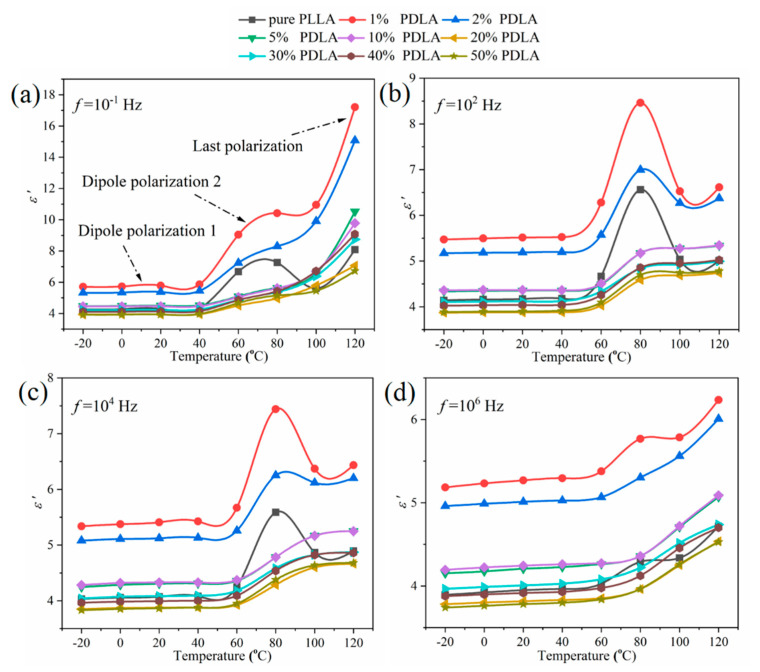
Temperature dependence of the dielectric constant of pure PLLA and PDLA/PLLA blends with different PDLA contents on the (**a**) 10^−1^, (**b**) 10^2^ (**c**) 10^4^, and (**d**) 10^6^ Hz, respectively.

Dependence of the dielectric constant (*ε*’) of pure PLLA and PDLA/PLLA blends on frequency and temperature is shown in Figure 6. The *ε*’ of pure PLLA is relatively low at low temperature and frequency, which is because chains and segments in the polymer are in a frozen state at low temperature and frequency, and cannot move along the direction of the electric field [20]. However, a weak dipole polarization caused by polar groups on the segments can undergo small angular deflection, even at low temperature and frequency of dipole polarization 1, as shown in Figure 6a. With addition of a small amount of 1% and 2% PDLA in PLLA, there is an obvious increase of *ε*’ due to presence of interface polarization. Dipole polarization 1 can still be detected at low frequency and temperature. This is because some polar groups on the segments (or the polar groups on the segments at a distance from the SC crystals) that are not affected by the SC crystals can still undergo small angle deflection. Notably, with an increase in PDLA content, the *ε*’ of the blends decreases and the dipole polarization peak of the blends disappears gradually, indicating that SC crystals have formed which restrain dipole polarization and reduce interface polarization. It is confirmed that SC crystals in the PDLA/PLLA blends possess the typical cross-linking network, as reported by [21].

Furthermore, as shown in Figure 6a, with an increase in test temperature up to glass transition temperature, a large polarization peak is observed in the curve of *ε*’ of pure PLLA, which is represented as dipole polarization 2, as the dipole polarization caused by polar groups and segments on the chains can be oriented along the electric field direction within this temperature range. With a further increase in temperature, the segment orientation along the electric field direction is destroyed due to the disordering action of molecular thermal motion, resulting in decrease of *ε*’. However, at temperature of >100 °C, each segment in the chains absorbs sufficient heat energy to drive movement of the entire chains, leading to chain reorientations along the electric field direction and making the *ε*’ of pure PLLA begin to increase again rapidly, which is represented as the last dipole polarization. In contrast, introduction of PDLA affords more compact chains in spatial structures of the blends. These tightly packed chains in the blends inhibit segment torsion, resulting in low apparent polarization, even at glass transition temperature or high temperature.

In general, formation of the last polarization process is also closely related to the beginning of the cold-crystallization process of the polymer, as it tends to occur in amorphous regions of the polymer. The degree of crystallinity of pure PLLA is very low, and numerous amorphous chains exist in pure PLLA, resulting in the *ε*’ of pure PLLA increasing rapidly. For PDLA/PLLA blends, formation of SC crystals greatly improves the degree of crystallinity of PDLA/PLLA blends, resulting in a decrease in amorphous regions. Meanwhile, loop chains, bound chains, or chips connected to the amorphous region are also inhibited by the physical cross-linking network in the SC crystals. As a result, the last polarization process does not change with an increase in the test temperature. In addition, dipole polarization peaks of pure PLLA and PDLA/PLLA blends reduce in intensity with an increase in the test frequency (Figure 6b–d). It is shown that at low frequency, polar groups have sufficient time to use the external electric field to establish polarization, but with an increase in frequency, the time required for polar groups to establish polarization is longer than the time required for alternating field oscillation, such that dipole polarization peaks decrease or disappear.

**Figure 7:**
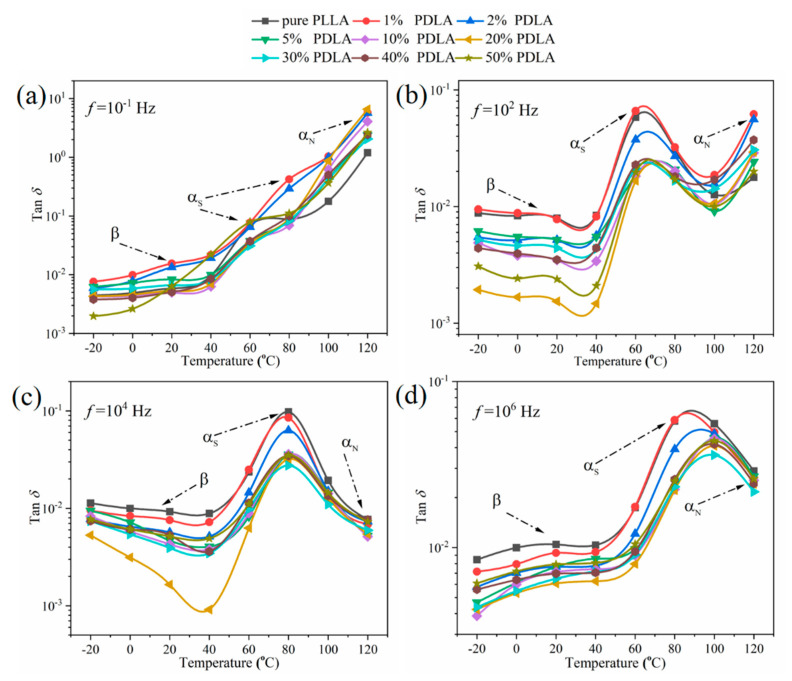
Temperature dependence of dielectric loss of pure PLLA and PDLA/PLLA blends with different PDLA contents on the (**a**) 10^−1^, (**b**) 10^2^, (**c**) 10^4^, and (**d**) 10^6^ Hz, respectively.

Dielectric loss (tan*δ*) reflects the relaxation process and mode of chain movement of polymer materials at different temperatures and frequencies. As shown in Figure 7, pure PLLA and PLDA/PLLA blends with different PDLA contents exhibit three relaxation processes, *β*-, *α*_S_-, and *α*_N_-relaxation, with an increase in temperature [22]. *β*-relaxation is caused by local torsion of the polar groups on the segment of PLLA and/or PDLA/PLLA blends in the glass state. This is widespread relaxation that occurs at low temperatures. As thermal motion of polar groups is very low and segments are frozen, the corresponding relaxation time is long, with insufficient time to establish with the external electric field, so the tan*δ* is small. With an increase in temperature, the segments in the chains of pure PLLA and/or PDLA/PLLA blends start to absorb heat energy, causing polar groups on segments to begin a violent twisting motion. The corresponding relaxation time decreases, which leads to a rapid relaxation process being established and an increase in tan*δ*. When the temperature is increased to the glass transition temperature, pure PLLA and/or PDLA/PLLA blends exhibit a glass/rubber state transition. The relaxation process of the segments in the chains of the pure PLLA and/or PDLA/PLLA blends is fully established, and a maximum value of tan*δ* is obtained. As orientation of polar groups cannot keep up with the change of electric field with an increase in temperature, the tan*δ* decreases. The complete relaxation process described above is called *α*_S_-relaxation, which is caused by related segment motion accompanied by a change in glass transition temperature. The *α*_S_-relaxation processes of pure PLLA and/or PDLA/PLLA blends show a tendency to shift toward high temperature with an increase in frequency, which may be related to an increase in ionic conductivity or electronic polarization, as shown in Figure 7b–d. When temperature is increased to >100 °C, strong chains of thermal motion in pure PLLA and/or PDLA/PLLA blends inhibit movement of segments along the direction of the electric field, such that the corresponding value of tan*δ* increases exponentially. This is the relaxation mode caused by the end-to-end vector fluctuation of the chains, which is called *α*_N_-relaxation.

Furthermore, it is found that peaks of *α*_S_-relaxation of the blends move toward high temperature with an increase in PDLA content at the same frequency. Owing to introduction of PDLA, the conformation rearrangement occurs in the main chain of macromolecules, resulting in formation of new hydrogen bonds and SC crystals, which hinder movement of polar groups on the segments. Interestingly, when the content of PDLA exceeds 10%, the *α*_S_-relaxation peak of PDLA/PLLA blends do not continue to shift toward high temperature due to formation of a physical cross-linking network in the SC crystals. In addition, *α*_N_-relaxation, which occurs at high temperatures, is not detected. It is considered that molten crystalline polymers usually have lamella structures. The amorphous region between the two layers is composed of a loop chain, tie chain, and dangling chains. As the ends of these sub-chains are fixed to the crystal lamella, no *α*_N_-relaxation in loops and tie chains is encountered. The relaxation time of the normal mode of cilia chains depends on the molecular weight between the free end and the end fixed on the surface of the lamella [23]. Introduction of PDLA can improve the degree of crystallinity of blends and decrease the amorphous region of blends as well as the molecular weight of cilia chains, such that *α*_N_-relaxation cannot be detected [24].

### 3.5. Tensile Properties and Fracture Behavior of the Blends

**Figure 8:**
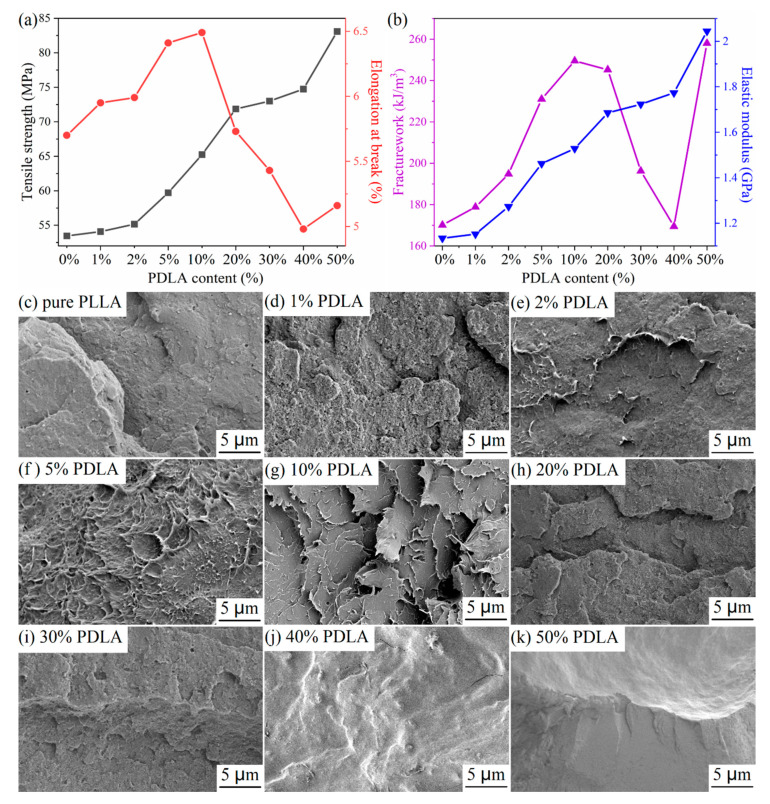
(**a**) Tensile strength/elongation at break, (**b**) fracture work/elastic modulus, and (**c**–**k**) FE-SEM images of the tensile fracture surfaces of pure PLLA and PDLA/PLLA blends with different PDLA contents.

The tensile properties of pure PLLA and PDLA/PLLA blends are shown in Figure 8a,b. Tensile strength, elongation at break, fracture work, and elastic modulus of pure PLLA are 52.98 MPa 5.71%, 170.10 kJ/m^3^, and 1.13 GPa, respectively. It is found that introduction of PDLA improves significantly tensile strength and the elastic modulus of the PDLA/PLLA blends. When the mass ratio of PLLA and PDLA is 1:1, that is, the blend is composed of the overall SC-PLA, tensile strength and the elastic modulus are up to 83.70 MPa and 2.04 GPa, respectively. It is thought that SC crystals with hydrogen bonds (C=O^…^H_3_C) force chains in PDLA/PLLA blends to closely align together, resulting in increases in tensile strength and the elastic modulus of the PDLA/PLLA blends. Changes in tensile strength and the elastic modulus can also be explained based on changes in the degree of crystallinity of PDLA/PLLA blends. Formation of SC crystals increases the crystallization capacity of PDLA/PLLA blends, resulting in increase in the degree of crystallinity of PDLA/PLLA blends. A high degree of crystallinity improves symmetry and regularity of the chain structure, thereby increasing tensile strength and the elastic modulus of the blends.

The elongation at break of the PDLA/PLLA blends shows a decreasing trend. Interestingly, when a small amount of PDLA (<10%) is blended with PLLA, the elongation at break of the blends tends to increase slightly, whereas at high PDLA contents (>10%) the elongation at break decreases. Morphologies of the tensile fracture of the PDLA/PLLA blends confirmed this result. In Figure 8c, the surface of tensile fracture of pure PLLA is smooth and neat, which is a brittle fracture characteristic. With an increase in PDLA content, some burrs begin to appear on the surface of the tensile fracture of the PDLA/PLLA blends, indicating that the tensile fracture characteristic of the PDLA/PLLA blends begins to change from brittle fracture to toughness fracture. With further increase in PDLA content, the surface of the tensile fracture of the PDLA/PLLA blends becomes smooth, indicating that fracture characteristics of the PDLA/PLLA blends again change from toughness to brittle fracture. The calculated fracture work of the PDLA/PLLA blends also showed a decreasing trend, followed by increasing and subsequent decreasing trends, as observed in Figure 8b. However, fracture work of the overall SC-PLA is higher than those of the other PDLA/PLLA blends, which is attributed to significantly higher tensile strength of the overall SC-PLA than those of the other PDLA/PLLA blends, while elongation at break does not change significantly, such that fracture work of the overall SC-PLA is very high.

## 4. Conclusions

In this study, the overall SC-PLA is successfully prepared via a melt-blending method using 1:1 mass ratio of PDLA and PLLA under adjusting processing condition. The degree of crystallinity of the overall SC-PLA is up to 43.7%, and its tensile strength, elastic modulus, elongation at break, and fracture work are 83.70 MPa, 2.04 GPa, 5.16%, and 258 kJ/m^3^, respectively. SC crystals formed in PLLA with a small amount of PDLA are not enough to form a cross-linking network in the blends and can be used as a nucleating agent to improve the degree of crystallinity of PLLA. In blends with high PDLA content (≥10%), formation of a physical cross-linking network in SC crystals can transform the molecular chain from a linear structure to a branched cross-linking structure, making the blends have a special microstructure. In addition, formation of a physical cross-linking network in the SC crystals is confirmed by dielectric spectroscopy, and interactions between HC crystals, SC crystals, and the amorphous region in blends were also analyzed. The above research on crystallization, and dielectric and mechanical properties of PDLA/PLLA blends provides a strong theoretical basis for subsequent processing and manufacturing of PLA.
